# Some Things for the Future

**Published:** 1916-06

**Authors:** 


### Some Things for the Future.

The Texas Medical Journal is a bit proud of the many good things it is offering its patrons these days. The editor may be just a little inclined to look with too much favor on the contents of the JOURNAL, but certainly its readers are not thus predisposed in its favor. Many of them write to say that they find this the most helpful of all the medical journals they read. It is because it is edited with a view to affording the greatest possible help on practical subjects that are attracting the attention of physicians and surgeons right at this time. The editor does not pretend to know how to write such articles, but has spent many years in the closest study of the one problem of finding the subjects of most vital interest to the profession and then finding the men who are qualified to write instructively on these subjects. The editors of most medical journals make editorial work a side issue; this editor makes it a life work. Perhaps that accounts for whatever superiority may be found in this publication.

Dr. Harrower's learned articles on "Internal Secretory Disorders" are worth to any wide-awake physician many times the cost of a year's subscription to this JOURNAL; in fact, they are invaluable to the man who has any desire to keep up. They will be a feature of the TEXAS MEDICAL JOURNAL for the next year.

Insanity is increasing at such an alarming extent that physicians everywhere are studying it closely, especially in its incipient stages. A better understanding of the subject is certainly to be desired, for the skillful physician who thoroughly understands the early diagnosis of this dread disease can often check it in its incipiency, when delay makes a cure slow and often impossible. A certain social stigma attaches to the insane patient who has to be sent away to an asylum for the insane, and this is felt by every member of the patient's family. What is it not worth to the family physician to be able to so handle even one case that this humiliation may be avoided? Dr. White's articles to appear in the TEXAS MEDICAL JOURNAL will tell you how an insanity expert makes a diagnosis of insanity in its incipient stages.

Pellagra is proving to be the scourge of the South, and is spreading to the North rapidly. Right now it is as much to be dreaded as tuberculosis,—perhaps more so, for the cause and treatment of tuberculosis are coming to be well understood, while pellagra is still baffling all efforts to stop its spread. The TEXAS MEDICAL JOURNAL has arranged with a number of pellagra specialists for articles on pellagra, and from the multitude of counsel hopes to be of valuable service to every physician interested in this subject.

Auto therapy, serum and vaccine diagnosis and functional tests and many other subjects now attracting general attention will be discussed during the next year, arrangements already having been made for papers from those who have made special studies of the various subjects.

It may interest you to know that the subscription list of the JOURNAL is growing now more rapidly than at any time before in its history. It is not uncommon to receive from fifty to a hundred new subscribers a week, which is proof that the efforts of the publisher are appreciated. With the specially good offerings for the next year, it is confidently expected that the subscription list will more than double before the end of the year. The publisher is going to keep the standard so high that the TEXAS MEDICAL JOURNAL will exceed every other Southern medical journal in circulation. This may sound boastful, but it is the mark toward which aim is being made, and nothing is going to be left undone that can be done to reach it.

People accomplish in this world about what they try hard enough to do, and if earnest, honest effort counts in the publishing business as in other things the TEXAS MEDICAL JOURNAL will be made all that could be expected of any medical journal in the great South, which has professional problems that can be handled successfully only by a Southern publication.

---

